# Preventive strategies to reduce the rate of periprosthetic infections in total joint arthroplasty; a comprehensive review

**DOI:** 10.1007/s00402-024-05301-w

**Published:** 2024-04-18

**Authors:** Omer Faruk Egerci, Aliekber Yapar, Fırat Dogruoz, Huseyin Selcuk, Ozkan Kose

**Affiliations:** Department of Orthopedics and Traumatology, Antalya Training and Research Hospital, Antalya, Turkey

**Keywords:** Periprosthetic joint infections, Total knee arthroplasty, Total hip arthroplasty, Prevention

## Abstract

The increasing frequency of total hip (THA) and knee arthroplasties (TKA) is marred by the rise in periprosthetic joint infections (PJIs) and surgical site infections (SSIs), with PJIs incurring costs over $1.62 billion as of 2020 and individual case management averaging $90,000. SSIs additionally burden the U.S. healthcare economy with billions in expenses annually. PJI prevalence in primary THA and TKA ranges from 0.5% to 2.4%, spiking to 20% in revisions and representing 25% of TKA revision causes. Projections estimate up to 270,000 annual PJI cases by 2030. Often caused by gram-positive bacteria, particularly methicillin-resistant staphylococci, these infections demand preventive measures. This review dissects PJI prevention across preoperative, intraoperative, and perioperative phases, aligning with evidence-based CDC and WHO guidelines. Preoperative measures include managing diabetes, obesity, tobacco use, Staphylococcus aureus screening and nasal decolonization, nutritional optimization, and management of inflammatory arthropathies. Intraoperatively, antibiotic prophylaxis, skin preparation, operative room environmental controls, surgical technique precision, and irrigation options are scrutinized. Perioperative concerns focus on anticoagulation, blood management, and infection risk mitigation. Integrating these strategies promotes a patient-centric care model, aiming to reduce PJI incidence, improve patient outcomes, and increase care cost-effectiveness in joint arthroplasty.

## Introduction

The increasing incidence of total hip and knee arthroplasties (THA and TKA) is shadowed by a worrying spike in periprosthetic joint infections (PJI) and surgical site infections (SSI). These complications pose serious health risks and impose financial strains, with PJIs alone costing over $1.62 billion as of 2020 [1] and individual case management reaching $90,000 [2]. Moreover, SSI repercussions ripple across the U.S. healthcare economy, costing billions each year. PJI affects a considerable proportion of primary THA and TKA patients, recorded between 0.5% and 2.4%, and is even more frequent in revision cases, reaching up to 20% [3, 4]. Specifically, infections account for 25% of TKA revisions, underscoring their prominence as a postoperative complication [5]. Projections indicate a startling escalation to potentially 270,000 annual PJI cases by 2030 in the U.S., emphasizing the urgency of the issue [1, 2]. On the microbiological front, gram-positive bacteria, notably staphylococci, are the usual suspects in PJIs, and their methicillin-resistant variants complicate treatment [6]. Evidence-based strategies that could potentially prevent approximately half of SSIs are crucial; comprehensive guidelines published by the CDC and WHO serve as invaluable navigational tools in this effort. The multifactorial etiology of PJI necessitates a multidimensional approach for its Prevention, mandating strategies that transcend the boundaries of traditional medical management. This comprehensive review dissects the preventive measures against PJI along the chronological phases of patient management: preoperative, intraoperative, and perioperative stages. Each phase is scrutinized through the lens of evidence-based medicine, highlighting the symbiotic relationship between systemic patient health, microbial ecology, and operative technicalities in the genesis and Prevention of PJI.

## Preoperative considerations

The preoperative phase is critical in setting the stage for favorable surgical outcomes, necessitating an evaluation of systemic pathologies and physiological aberrations that could potentiate the risk of PJI. We delve into the management of chronic conditions such as diabetes mellitus and its glycemic control, the implications of obesity, and the systemic and local consequences of tobacco use. Additionally, we underscore the necessity for Staphylococcus aureus screening and subsequent nasal decolonization, both instrumental in reducing the microbial burden. A discourse on optimizing nutritional status to circumvent the pitfalls of malnutrition and managing inflammatory arthropathies to reduce perioperative immunological compromise is also presented.

## Intraoperative strategies

Transitioning to the intraoperative arena, our focus shifts to elements within the surgical milieu. We begin with exploring antibiotic prophylaxis, emphasizing both the selection and timing, followed by a discussion on the surgical site skin preparation protocols. The review addresses operative room environmental controls, including sterilization practices and airflow systems, to mitigate the risk of contaminant exposure. Surgical techniques, particularly the surgeon's approach, are dissected to elucidate the role of surgical precision in infection prevention. Intraoperative irrigation options are discussed, providing insights into the optimal solutions and techniques for reducing microbial presence.

## Perioperative modalities

The perioperative period, bridging the pre- and post-surgical phases, demands a holistic approach to patient management. Herein, we evaluate anticoagulation strategies, balancing the risk of thromboembolism with the potential for bleeding complications, both of which can influence infection rates. Blood management is examined in the context of both conservation strategies and transfusion practices, given the substantial role of optimal hemostasis and oxygenation in surgical recovery and infection prevention.

In synthesizing these components, this review illuminates the intricate tapestry of factors influencing PJI incidence. By integrating a spectrum of preventive strategies across the preoperative, intraoperative, and perioperative phases, we advocate for a more robust, patient-centric model of care. Such an approach, enriched with interdisciplinary collaboration, promises a strategic front in mitigating PJI risk, optimizing patient outcomes, and enhancing the cost-effectiveness of care in the domain of joint arthroplasty.

## Preoperative considerations

### Diabetes mellitus

Diabetes mellitus, a global health concern, is increasing in prevalence. It is estimated to affect approximately 9.3% of the adult population worldwide and is projected to reach a staggering 44 million individuals in the United States within the next two decades [7]. As total joint arthroplasty (TJA) procedures become more commonplace [8], managing diabetes-related complications is of paramount importance, especially given the rise of periprosthetic joint infections (PJI). In a meticulous meta-analysis by Ren et al., including 3,561,446 hip arthroplasty cases across Asia–Pacific, Europe, and the USA, a significant correlation was found between diabetes mellitus and an increased risk (with a relative risk of 1.64) of periprosthetic joint infection (PJI) following primary total hip arthroplasty (THA) [9]. This risk is further emphasized in knee replacement surgery. Drawing from 18 studies covering 119,244 individuals and 120,754 knee procedures, diabetic patients undergoing total knee arthroplasty demonstrated a risk of infection 1.84 times greater than their non-diabetic counterparts (registering infection rates of 1.9% for diabetics versus 1.2% for non-diabetics), coupled with a 1.96 times increased risk of deep surgical site infection. [10].

Diabetes mellitus is intricately linked with a higher likelihood of surgical site infections. Specifically, uncontrolled diabetes, defined by blood glucose levels surpassing 200 mg/L or Hgb A1c values greater than 7%, can notably amplify the risk of postoperative complications. The American Diabetes Association has recommended delaying surgery for those with an HbA1c greater than 7% [11]. These complications can be attributed to several factors. The long-term effects of hyperglycemia compromise the immune system and microangiopathic changes impede wound healing in diabetic patients.

Hemoglobin A1c (Hgb A1c) serves as a frequently used metric for long-term glycemic control, ideally maintained below 7.0%, translating to an average glucose concentration of about 154 mg/dL [12]. However, perioperative glucose levels have emerged as even more indicative of periprosthetic joint infection (PJI) risks than Hgb A1c alone [13]. Stress from surgical procedures can induce hyperglycemia, not just in diabetic patients but also in those without a prior diabetes diagnosis, thereby independently escalating the risk for infections. Hence, it is highly recommended to maintain perioperative blood glucose concentrations between 110 and 180 mg/dL, facilitated by routine monitoring and adept postoperative diabetic management protocols (14). Considering the recent evidence, meticulous preoperative optimization has become the standard protocol, especially as over 30% of TJA patients harbor undiagnosed hyperglycemia [15]. Various factors such as undergoing revision TJA, obesity, male gender, and fluctuations in glucose levels play into this risk. Both the World Health Organization (WHO) and the Centers for Disease Control and Prevention (CDC) underline the importance of rigorous glycemic control during surgery, irrespective of a patient's diabetic status.

While HbA1c remains a conventional tool for ascertaining glycemic control, its reliability has been challenged in recent years. Fructosamine has emerged as a potential replacement for this role, especially with values exceeding 293 mmol/L being associated with heightened risks of surgical site infections, subsequent surgical interventions, and hospital readmissions [16].

Promising developments in the realm of glycemic control markers, such as the advent of glycated albumin (GA), are on the horizon. Compared to HgbA1c and fructosamine, GA demonstrates greater sensitivity and standardization [17]. A multicenter prospective study is currently in progress to further delineate the prognostic utility of GA for patients undergoing primary TJA [18].

Given the pivotal connection between diabetes mellitus, hyperglycemia, and complications following TJA, it is essential to strike a balance. The risks associated with postoperative infections in diabetic patients and perioperative hyperglycemia must be weighed against the potential improvements in the quality of life that TJA offers. Consequently, the call for patient optimization takes center stage, requiring judicious contemplation of the multifaceted interplay between diabetes management, the timing of the surgical procedure, and the prospective benefits of TJA.

### Obesity

The burgeoning prevalence of obesity presents a critical challenge in the realm of orthopedic surgery, particularly in the context of prosthetic joint infections (PJI). Patients undergoing total joint arthroplasty (TJA), such as total hip arthroplasty (THA) and total knee arthroplasty (TKA), are especially vulnerable to the risks obesity can impose. Extensive meta-analysis has demonstrated that a high body mass index (BMI) significantly elevates the risk of PJI, with patients boasting a BMI greater than 40, 35, or 30 facing 2.99-fold, 2.46-fold, or 2.02-fold higher risks, respectively, compared to individuals with lower BMIs, a finding consistent with recent studies. [9, 19]. Furthermore, morbid obesity (BMI > 40 kg/m^2^) has been shown to increase the risk of infection up to 21 times, specifically affecting outcomes post TKA [20]. The risk associated with elevated BMI is not linear; it increases exponentially along the BMI value scale [9]. Xu C et al. found that each one-unit increase in BMI was linked with an 8% higher risk of PJI [21]. This increased risk might be attributed to several factors, including longer surgical and anesthesia times, increased risk of wound colonization by bacteria such as C. avidum, and increased hospital stay and readmission rates [9, 22, 23]. Additionally, the poor vascularization of adipose tissue is another factor that compounds the risk of infection.

The adverse implications of obesity on TJA outcomes have been extensively researched. One study involving 97 hips undergoing two-stage revision surgery for PJI found a significantly lower implant survival rate at mean 5 years in both smokers and obese patients [24]. Another study revealed that the all-cause revision rate doubled in patients with a BMI of 35 kg/m^2 or greater and tripled in morbidly obese patients [9].

Obesity brings with it a constellation of other health risks that further complicate surgical outcomes. For instance, obese individuals often have a higher presence of metabolic syndrome, wound dehiscence, and heart disease. Similarly, a sub-group of obese patients with metabolic syndrome was found to be at an elevated risk of PJI [14].

Given these heightened risks, weight loss prior to TJA has been widely recommended. While setting a specific BMI cutoff as a goal may prove challenging for super-obese patients (BMI > 50), even a 5 to 10% weight reduction has been shown to provide cardiovascular and metabolic benefits [24]. In more severe cases, bariatric surgery has been considered a potent weight-loss strategy. However, the medical community remains divided on its efficacy as a preparatory measure for joint surgeries. Some studies argue that undergoing bariatric surgery before a joint replacement can significantly reduce postoperative complications, while others refute these claims, highlighting the need for more extensive research on the topic [24].

Further exacerbating the issue is the cyclical relationship between obesity and osteoarthritis. Several studies have shown that the prevalence of obesity has led to a corresponding increase in the rate of osteoarthritis, which in turn necessitates more frequent arthroplasty procedures [14]. Despite similar satisfaction and functional improvement in obese and non-obese patients, obese individuals are at a higher risk of PJI, surgical wound complications, wound dehiscence, hematoma formation, and prolonged wound drainage. A consensus by the American Association of Hip and Knee Surgeons (AAHKS) has suggested delaying total joint arthroplasty in patients with a BMI > 40, especially if associated with other comorbid conditions [14].

Preoperative optimization should be multidimensional, encompassing lifestyle changes and pharmaceutical interventions, where possible. For example, special attention should be given to antibiotic prophylaxis as inadequate dosing is not uncommon in the obese population. Furthermore, achieving a state of nutritional adequacy is crucial, as obese patients can often be paradoxically malnourished [25].

In conclusion, obesity remains a significant, modifiable risk factor for increased complications following TJA, including a heightened risk for PJI. Orthopedic surgeons should incorporate these considerations into their preoperative counseling and risk stratification for potential TJA candidates [22].

### Smoking

In the context of prosthetic joint infection (PJI) prevention, particularly following total joint arthroplasty (TJA), addressing modifiable risk factors like smoking is paramount. Smoking has been the subject of extensive research with mixed findings, yet the bulk of the evidence suggests that it contributes to increased risk of PJI and other surgical site infections (SSIs).

One meta-analysis indicated that smoking was not correlated with an elevated risk of PJI following total hip arthroplasty (THA). Specifically, the study revealed a pooled Relative Risk (RR) of 1.24 with a 95% Confidence Interval (CI) ranging from 0.85 to 1.82 for smokers who consumed > 45 g/day (males) or > 30 g/day (females). However, the study highlighted that smoking may produce a synergistic effect, elevating PJI risk by 3.54-fold among obese patients. [9].

However, more recent evidence indicates a strong link between smoking and adverse surgical outcomes. A 2022 meta-analysis involving 234,937 patients demonstrated that smoking significantly increased the risk of PJI, with an Odds Ratio (OR) of 1.84 and a 95% CI ranging between 1.52–2.24. The study underlined the detrimental effect of smoking on all phases of wound healing, making a solid case for its role as a modifiable risk factor for PJI [26]. Further supporting this result, a 2023 study presented at a JBJS meeting found that among 97 hips that underwent two-stage revision surgery for PJI, both smokers and obese patients had significantly lower implant survival rates, with a statistical significance level of p = 0.001 [27]. These conflicting results underscore the need for more robust trials to ascertain the precise relationship between smoking and PJI.

Several mechanisms have been proposed to explain the negative impact of smoking on surgical outcomes. Nicotine, the primary offending component of tobacco, has been linked to microvascular constriction and decreased oxygen delivery to tissues. These changes impair wound-healing, making post-surgical infections more likely [28]. Furthermore, smoking affects collagen synthesis and maturation, critical for tissue repair and contributes to vasoconstriction and hypoperfusion, thus increasing susceptibility to infection [29]. Smoking has also been shown to have immunomodulatory effects, disrupting immune cell function and neutrophil defense mechanisms against pathogens.

Preoperative optimization is considered essential for minimizing risks. Current Clinical Practice Guidelines (CPGs) recommend preoperative risk stratification that includes assessing modifiable risk factors like smoking alongside others such as obesity, diabetes, and hypertension [30]. Given that smokers undergoing TJA have shown significantly higher rates of surgical site infection compared to non-smokers [31], smoking cessation programs have been highly recommended.

Although the optimal duration for preoperative smoking cessation remains to be established, it's generally suggested that a minimum of 4–6 weeks of smoking cessation prior to surgery can be effective [32, 33]. Some reports have even advocated for more extended cessation periods of up to 6 months [31]. Smoking cessation can be confirmed through serum cotinine assays, and patients undergoing smoking cessation therapy using transdermal nicotine patches should be monitored as they continue to test positive for serum cotinine [23]

Despite the strong evidence supporting the benefits of preoperative smoking cessation, the question of whether smokers should be outright refused elective surgery remains controversial [27]. What is clear is that smoking remains a complex risk factor for PJI that requires further study. Until then, cautious optimism and a focus on preoperative optimization remain the best courses of action.

In summary, although there is some discrepancy in the literature regarding the risk of PJI attributed to smoking, the preponderance of evidence suggests a negative impact, particularly when combined with other risk factors like obesity. Hence, smoking cessation remains a recommended strategy in the preoperative period to minimize this risk.

### Staphylococcus aureus screening and nasal decolonization

In the specialized field of Total Joint Arthroplasty (TJA), Staphylococcus aureus and its methicillin-resistant variant (MRSA) are significant causes of concern when it comes to Prosthetic Joint Infections (PJIs). These infections have severe implications, ranging from prolonged hospitalization to multiple surgeries, significantly impacting the patient's quality of life. With an alarming prevalence rate of S. aureus, including MRSA, of up to 30% in patients undergoing TJA [34] the need for effective preoperative screening and decolonization measures has never been more crucial.

A common screening method involves the use of nasal swabs followed by rapid polymerase chain reaction (PCR) techniques. These tests have proven to be highly efficient in identifying colonized patients and facilitating timely interventions [35, 36]. After screening, physicians usually recommend decolonization procedures to patients who test positive for S. aureus or MRSA. Typically, the decolonization process involves the application of mupirocin nasal ointment twice daily to both nares and daily bathing with chlorhexidine for five days leading up to the scheduled surgery [23]. For those who test positive for MRSA, an additional intraoperative dose of vancomycin is often recommended [23]. This comprehensive approach lessens the risk of SSIs and translates to significant economic benefits for healthcare systems [37].

There has been some debate around the effectiveness of these preoperative screening and decolonization measures. While some meta-analyses have pointed to a beneficial effect of preoperative decolonization in reducing SSIs [9, 38–40], others have argued that these protocols have not effectively reduced SSI rates [41, 42]. Despite these conflicting reports, it is generally agreed that individuals colonized with S. aureus are at higher risk for developing SSIs, and thus some form of intervention is warranted [43].

Moreover, besides mupirocin, other topical antiseptics like povidone-iodine swabs and alcohol-based gels are considered promising in reducing S. aureus nasal carriage [43, 44]. Concerns about mupirocin resistance have been raised but short-term use for decolonization does not appear to significantly contribute to resistance [45, 46]. Furthermore, it has been reported that universal decolonization strategies could be as effective as screening-based methods, without a significant difference in SSI rates between the two [43].

Importantly, these preoperative protocols have economic benefits as well. Implementing an institution-wide screening program not only leads to a significant reduction in postoperative rates of SSIs but also results in economic gains for the hospital.

In summary, although some inconsistencies in research findings exist, current evidence strongly advocates for the utility of preoperative screening and decolonization protocols for managing and preventing PJIs in TJA. Nevertheless, to definitively settle these debates, more high-quality randomized controlled trials are warranted.

### Malnutrition

Nearly half of surgical patients face malnutrition, associated with complications like prolonged intubation, wound infections, higher mortality, and extended hospital stays [47]. Malnutrition diagnosis involves laboratory tests, including albumin levels under 3.5 g/dL, total lymphocyte counts under 1500 cells/mm3, and transferrin levels under 200 mg/dL [48]. Anthropometric indicators also contribute to the diagnosis, such as calf and arm muscle circumferences below 31 cm and 22 mm, respectively, and significant triceps skinfold presence [48]. Despite the existence of several nutrition scoring tools, like the Rainey-MacDonald nutritional index and Mini Nutritional Assessment, their validation is inconsistent [49]. While no definitive diagnostic standard for malnutrition exists, serological criteria remain the most prevalent and explored method [48].

In the context of preventing prosthetic joint infections (PJI), the role of nutritional status has garnered increasing attention. One extensive meta-analysis revealed a strong association between serologic malnutrition and a higher rate of postoperative complications following total joint arthroplasty (TJA), such as various types of surgical site infections. Specifically, the malnourished cohort had significantly increased risks for issues like wound disruption and both superficial and deep incisional surgical site infections [50], It's not just the visibly undernourished who are at risk. Paradoxically, obesity does not preclude malnutrition. Studies have found that as many as 42.9% of obese patients undergoing TJA are malnourished, often consuming diets that are high in calories but low in essential nutrients [51].

Identifying those at risk for malnutrition is a crucial step in Prevention. Simple and readily available laboratory tests like total lymphocyte counts, serum albumin levels, and transferrin levels can offer quick identification [52]. When malnutrition is identified, working with a dietician to improve nutritional status can prepare patients for the metabolic demands of the postoperative period [52].

Another dimension of nutritional preparedness involves Vitamin D, a vital nutrient for bone homeostasis. A high prevalence of Vitamin D deficiency has been identified in patients experiencing PJI. Therefore, Vitamin D levels should be checked preoperatively, and supplementation should be initiated if deficiencies are found [53].

Although the current evidence strongly supports the role of nutritional status in PJI risk, some limitations in existing studies must be noted. These include methodological shortcomings and a lack of demographic granularity such as age, gender, and ethnicity [50]. Furthermore, common indices used for diagnosing malnutrition are limited to identifying protein deficiencies and often overlook caloric and vitamin deficiencies [54]. Therefore, while malnutrition, whether serologic or paradoxical, substantially raises the risks associated with TJA—including the risk of PJI—more comprehensive approaches to identifying and treating malnutrition are needed. Preoperative nutritional assessment and appropriate interventions, including high-protein supplements and vitamin and mineral supplementation, are advised to improve surgical outcomes [55]. Given the severe implications of PJI, including longer hospital stays and substantial morbidity, future research should aim to validate these findings and explore the multifaceted impact of malnutrition on surgical outcomes.

### Inflammatory arthropathies

In the context of prosthetic joint infection (PJI) prevention, special attention must be given to patients with inflammatory arthropathies such as rheumatoid arthritis (RA) and systemic lupus erythematosus (SLE). These patients are already at an elevated risk for postoperative infections due to both the nature of their underlying conditions and the complex pharmacological regimens they follow, which often include immunomodulators and biologics [56–58].

One of the primary considerations for these patients involves the use of Disease Modifying Antirheumatic Medications (DMARDs), such as methotrexate. According to the guidelines jointly published by the American College of Rheumatology and American Association of Hip and Knee Surgeons, traditional DMARDs generally do not need to be withheld prior to surgery [59]. However, this consensus is not entirely universal. Some studies, like those by Carpenter et al., suggest that withholding methotrexate one week before and after surgery can actually decrease the risk of PJI in patients with RA [60].

Biological DMARDs, on the other hand, pose a more significant concern. These include medications that inhibit tumor necrosis factor alpha (TNF-α), a molecule that plays a pivotal role in immune responses but also can contribute to inflammation when overactive. Although these inhibitors, such as adalimumab and etanercept, are effective in managing conditions like RA, they also place patients at higher risk for opportunistic infections. Thus, current guidelines generally recommend withholding biologics one dosing cycle prior to elective total joint arthroplasty (TJA). They can be restarted once the surgical wounds have adequately healed and there is no sign of infection [58, 59].

The role of corticosteroids in the management of inflammatory arthropathies also poses unique challenges in the context of TJA. Corticosteroids like prednisone are known to suppress the immune system, thereby increasing the risk of PJI. Therefore, any patient undergoing TJA who is on corticosteroid therapy should be closely monitored for signs of infection postoperatively.

The literature presents some conflicting evidence, and there are varying guidelines from different organizations. For example, while the American College of Rheumatology and the British Society for Rheumatology recommend withholding TNF-α inhibitors around the time of TJA, the International Consensus Group (ICG) on Periprosthetic Joint Infection has developed its own set of cessation schedules for different immunosuppressive agents [61–63].

In summary, it is essential to individualize treatment plans for patients with inflammatory arthropathies undergoing TJA, considering both the underlying disease and the medications used for its management. The goal is to strike a balance between managing the underlying arthropathy and minimizing the risk of PJI, necessitating a multidisciplinary approach that involves rheumatologists, orthopedic surgeons, and infectious disease specialists.

## Intraoperative strategies

### Antibiotic prophylaxis

In the Prevention of prosthetic joint infection (PJI), the role of perioperative antibiotic prophylaxis is pivotal. Prophylactic antibiotic use has been validated as the most crucial factor in preventing both surgical site infections (SSIs) and PJIs [64]. It is widely recognized as a core component of a complex preventative strategy and is never intended to replace other preventive measures [65].

The general consensus across clinical practice guidelines (CPGs) and studies is that first- or second-generation cephalosporins, such as cefazolin, should be the antibiotics of choice due to their broad-spectrum antimicrobial activity, favorable bioavailability, and cost-effectiveness [66].

For patients who report allergies to beta-lactam antibiotics, alternatives such as clindamycin or vancomycin are recommended. Despite traditional hesitancy to use cephalosporins in those with penicillin allergies, current evidence suggests that the risk of cross-reactivity is low, especially in the absence of a documented anaphylactic reaction to penicillin [67]. On the other hand, in cases of high-risk patients, such as those in nursing homes an dialysis units, or those with a history of methicillin-resistant Staphylococcus aureus (MRSA) infection, health care professionals dual antibiotic prophylaxis consisting of a cephalosporin and either vancomycin or teicoplanin is recommended [68]. Teicoplanin has been reported as the most common agent used in high-risk patients in Europe [69].

Timing is a critical factor as well. The general consensus advocates for the administration of a weight-adjusted (15 mg/kg) antibiotic dose 30 to 60 min before the surgical incision for cephalosporins. For antibiotics like vancomycin, which require a longer infusion time, the infusion should ideally start 60 to 90 min before the incision [70]. Similar to cephalosporins, clindamycin should be administered within 60 min before the skin incision. [14]. It is further suggested the antibiotic dose should be repeated if surgery extends beyond 2 to 3 h or if there is substantial blood loss [71].

Regarding the duration of antibiotic therapy, most guidelines and studies support discontinuation within 24 h post-surgery. Prolonged use does not seem to offer additional benefits and is linked to the emergence of antibiotic-resistant strains [72].

Moreover, the use of local antibiotics, particularly vancomycin powder, as a prophylactic measure against prosthetic joint infections (PJI) in total hip arthroplasty (THA) and total knee arthroplasty (TKA) has garnered significant attention in recent years. This method theoretically allows for elevated local antibiotic concentrations, potentially diminishing PJI risk [73–75] and may reduce systemic adverse effects versus intravenous administration [76]. Nonetheless, existing research lacks the statistical power to definitively quantify side effects.

Despite certain studies and meta-analyses proposing that topical vancomycin could decrease PJI incidence [75, 77, 78], there is conflicting evidence with no definitive correlation to reduced infection rates and reported post-application complications [79–81]. Many current meta-analyses incorporate heterogeneous studies with varying evidence quality, potentially introducing significant bias and undermining the reliability of conclusions [75, 77, 78].

Additionally, topical antibiotic use is not without potential detriments, encompassing complications in wound healing, inhibited osteoblast function, third-body wear, and critically, the propensity to engender antibiotic resistance [82]. Even with diminished systemic absorption, the risk persists for reactions such as allergies, ototoxicity, and nephrotoxicity [82, 83], not to mention the serious broader implication of promoting resistant microbial strains.

A 2021 comprehensive systematic review assessed 2408 studies, ultimately focusing on nine encompassing 3371 subjects who underwent vancomycin treatment juxtaposed with 2884 untreated individuals. It concluded a conspicuous absence of definitive efficacy evidence, coupled with an insufficient safety profile, suggesting that the routine integration of topical vancomycin in THA and TKA is unwarranted [84].

Concisely, prevailing studies insufficiently advocate for topical vancomycin as a standard prophylactic measure in THA and TKA, emphasizing the requisite for more comprehensive, stringently conducted trials to confirm its therapeutic validity and safety spectrum.

In the context of revision cases, although conventional guidance advises against the use of antimicrobial agents for a minimum of two weeks before culture sampling to reduce the likelihood of obtaining false-negative outcomes some emerging evidence from a small-scale study indicates that preoperative antibiotic prophylaxis may not compromise the reliability of these tissue cultures [85, 86].

Notably, despite established guidelines, non-compliance rates can be as high as almost 40%, especially in relation to dose adjustments by weight and the timing of prolonged treatment. Such non-compliance has been associated with a higher risk of SSI [71].

In conclusion, the judicious use of antibiotic prophylaxis remains crucial for mitigating the risks of PJI and SSIs. It's imperative for healthcare providers to stay current with evolving guidelines on antibiotic selection, dosing and timing to optimize patient safety and surgical outcomes.

### Skin preparation

In the context of preventing prosthetic joint infection (PJI), the importance of preoperative skin preparation is well-acknowledged. The primary aim is to substantially decrease the bacterial load on the patient's skin, which could otherwise serve as a source for postoperative infection [87, 88]. The choice of antiseptic agents has generated considerable debate, given conflicting perspectives on their efficacy.

Chlorhexidine gluconate-based solutions have garnered widespread support for surgical site preparations. The solution disrupts bacterial cellular membranes and its antimicrobial effect is longer-lasting compared to iodophors. Additionally, preoperative bathing with chlorhexidine-impregnated cloth the night before and the morning of the surgery has shown promise in decreasing the rate of PJI [89].

On the other hand, one study indicates iodine may be superior to CHG in PJI prevention [90, 91]. Moreover, there are concerns regarding the effective concentration of chlorhexidine, as bacterial cultures could still be obtained even after applying a 4% chlorhexidine solution [92].

To bridge these gaps, a combined approach involving chlorhexidine gluconate and isopropyl alcohol is often recommended [93]. Alcohol, though lacking residual activity, acts as a potent antimicrobial agent and complements the effects of chlorhexidine. Dual preparation of the skin has been advised, as contamination can occur during the draping process [93].

Regarding the practice of hair removal at the surgical site, the traditional belief was that it helps in reducing the risk of surgical site infection (SSI). Thus, the Centers for Disease Control and Prevention now advocate for using electric clippers if hair removal is considered necessary [87]. While the benefits of preoperative skin decontamination are clear, care should be taken to avoid excessive or aggressive skin preparation. Such practices could inadvertently compromise the integrity of the superficial skin layers, thereby paradoxically increasing the risk of infection [87].

In summary, although chlorhexidine gluconate often gets the nod for its prolonged antibacterial action, the optimal antiseptic solution could involve a combination of agents, including alcohol, to provide a broad-spectrum antimicrobial effect. Nonetheless, it's important to note that the methodology of many comparative studies leaves room for improvement. Hence, further research is essential to establish the most effective skin preparation protocol for the Prevention of PJI.

### Operation room

Prevention of prosthetic joint infection (PJI) requires a multi-pronged approach that includes various factors such as operating room environment, personnel behavior, and surgical practices. A comprehensive understanding of these factors is essential for reducing the rate of surgical site infections (SSIs), particularly in total joint arthroplasty (TJA).

Firstly, the operating room environment is critical for infection control. Intraoperative wound contamination is predominantly due to airborne pathogens, most of which originate from the surgical team itself [94]. Limiting operating room traffic has been shown to significantly reduce the rate of SSIs [95, 96]. Particularly for arthroplasty procedures, it is imperative to restrict the number and movement of personnel near the operating theatre [95, 96]. Studies have found a strong correlation between microbial load in the operating room and the number of personnel present [97, 98].

Various modifications to the OR environment have been examined, such as ultraviolet light, laminar flow systems, and body exhaust suits [95, 96].

The effectiveness of laminar air flow systems in reducing bacterial load within the operating room has been supported by multiple studies [71, 94], holding promise for improved infection control. However, the translation of reduced bacterial load into consistent clinical outcomes, specifically in terms of surgical site infections (SSIs) or prosthetic joint infections (PJIs), remains inconsistent. Notably, a significant discrepancy in PJI rates between operating rooms equipped with laminar air flow systems and those without was not observed in a substantial Asian population study [99]. The complex consideration of economic factors further complicates the assessment, as the cost-effectiveness of these systems is debatable. While they do exhibit the capacity to lower bacterial load, the potential advantages may not be economically justified due to substantial initial investments and ongoing maintenance expenses [100, 101]. Similarly, the use of body exhaust suits is controversial, and their efficacy is not universally supported [102, 103]. Ultraviolet lights can decrease viable bacteria but are not recommended due to potential harm to OR personnel [104].

Interestingly, guidelines show a level of caution in strongly recommending these systems. The World Health Organization (WHO), based on low-quality evidence, recommends that laminar air flow should not be used [105]. The Centers for Disease Control and Prevention (CDC) have not issued explicit recommendations on this issue [106].

To maintain a sterile environment, professional behavior among all operating room personnel is critical. National and international guidelines have checklists and protocols that need to be adhered to [107]. Adherence to these protocols is essential, even though achieving 100% compliance is challenging.

Hand antisepsis stands out as the most cost-effective measure in the operating room. Both alcohol-based hand rubs and water-based hand scrubs with certified antiseptics have been employed, though neither has proven to be significantly superior in reducing SSIs [108]. Surgical team members should wear appropriate gowns since even sterile surgical gloves and gowns can become contaminated during extended procedures, with double gloving particularly recommended for TJA surgeries to reduce contamination [109]. Many surgeons also change gloves multiple times depending on the length and phase of the surgery.

The use of skin sealants and drapes at the incision site has also been a subject of discussion. Newer drapes are often imbued with bacteriostatic agents like iodine or chlorhexidine with the aim of mitigating bacterial activity and contamination at the incision site during surgery [110]. Despite this, the World Health Organization and the Centers for Disease Control and Prevention have not firmly endorsed draping the area based on existing evidence [71]. Still, some contemporary research studies do advocate for the use of these drapes, suggesting they may indeed offer some protection against SSIs [111, 112].

In conclusion, Prevention of PJI necessitates a disciplined approach to operating room procedures and personnel conduct. While technological interventions such as laminar flow and body exhaust suits have not consistently proven their effectiveness, traditional methods like limiting OR traffic, hand antisepsis, and double gloving remain significant for mitigating infection risks.

### Surgeon’s approach

The Prevention of Prosthetic Joint Infections (PJIs) is a complex interplay of numerous factors, including the surgical technique, wound management, and time spent in the operating room. Surgeons play an instrumental role in optimizing these variables to minimize the risk of postoperative infections.

In the context of wound closure, the available evidence does not point towards a universally superior method, but rather suggests that the choice of technique and materials should be tailored to individual cases and specific types of surgeries, such as Total Knee Arthroplasty (TKA) [113]. The tension applied during wound closure needs to be delicately balanced; too much can lead to skin necrosis, while insufficient tension might not adequately counteract mechanical forces on the wound. The utilization of non-absorbable sutures and the excessive use of electrocautery can raise infection risks, and therefore, should be used judiciously [114]. Subcutaneous suture lines can help in significantly reducing tension on skin sutures, thereby preventing excessive wound tightness. Strategies like using subcuticular sutures along with skin adhesives or silver-impregnated occlusive dressings are showing promise in lowering the risk of superficial drainage and subsequent infection [115, 116].

The duration of the surgery is another critical variable. Multiple studies have demonstrated that each additional hour in surgery can increase the likelihood of SSI by up to 37% [117]. This is not only because of the greater opportunity for bacterial contamination but also because the efficacy of prophylactic antibiotics can wane over time, requiring re-dosing. However, it's worth noting that shorter surgical times should not compromise the quality of the procedure; complexity and the necessity for meticulous care can inevitably extend time in the operating room [118]. The data suggests that experienced surgeons who can operate both quickly and efficiently have lower PJI rates, underscoring the value of skill and experience in this context [119].

Handling of soft tissues during surgery also has implications for PJI risk. Surgeons need to ensure that soft tissues are manipulated with sterile instruments rather than hands or potentially contaminated gloves and the size of the surgical incision should be large enough for adequate visualization but not so large that it places undue stress on surrounding tissues [71]. Meticulous clotting of blood and tissue-preserving techniques are beneficial in leaving periarticular tissues vital without excessive hematomas.

In summary, surgeons can exert significant influence on the risk of PJIs through their choices in wound closure techniques, the speed and efficiency of their work, and their handling and respect of tissues. While it's challenging to pinpoint a single best practice given the multifactorial nature of PJIs, a composite approach that incorporates the best available evidence in each of these domains is likely the most effective strategy. Therefore, ongoing education, skill development, and adherence to evolving best practices are crucial for surgeons in their role in preventing PJIs.

### Irrigation options

In the Prevention of Prosthetic Joint Infections (PJI), one of the most controllable factors in a surgical setting is the use of antiseptic irrigation solutions prior to wound closure. Not only does this serve to physically remove debris, but it also acts to chemically and biologically reduce the bacterial load in the wound, thereby diminishing the risk of postoperative infection.

Povidone-iodine (PVP-I) has been widely endorsed as a preferred antiseptic solution. At many institutions, including the one cited by Parvizi et al., a 0.5% PVP-I solution is standard protocol due to its proven efficacy and low fibroblast toxicity [71, 120]. A systematic review and meta-analysis encompassing a total of 63,950 patients further corroborates the effectiveness of povidone-iodine (PI). This study revealed that PI reduced postoperative infection rates when compared to normal saline, with an odds ratio (OR) of 0.44 and a 95% confidence interval (CI) between 0.34 and 0.56. However, the efficacy of PI was found comparable to that of chlorhexidine gluconate (CHG), presenting an OR of 1.61 and a 95% CI of 0.83–3.09 [90].

The concentration of PVP-I used can also be critical. Hart and Hernandez in their retrospective studies observed a non-significantly higher infection rate with 0.25% PI when compared with non-PI agents. These findings raise concerns about the ideal concentration to be used, especially when most of the other research relies on a 0.35% concentration [121, 122].

Betadine, another iodine-based solution, is also frequently used. For instance, Brown et al. reported significant improvements in infection rates after using dilute Betadine lavage before surgical wound closure. This procedure has been recommended to consist of a 3.5% solution in 500 cc of normal saline, applied for three minutes, and then thoroughly washed out with normal saline [123].

Chlorhexidine offers an alternative to iodine-based solutions, with at least one study finding it non-inferior to dilute Betadine wash [124]. Moreover, it has been effective at concentrations as low as 2% against MRSA biofilm [125].

Detergents present another category of irrigation solutions. Substances like castile soap or benzalkonium chloride have proven effective in disrupting bacterial adherence properties, making them superior to saline and antibiotic solutions in vitro [126].

The addition of antibiotics to irrigation solutions remains contentious. Although in vitro studies have suggested some effectiveness, concerns remain about the potential for wound complications, dermatitis, hypersensitivity reactions, and bacterial resistance [127].

As for the technique of irrigation, high-pressure pulsatile lavage offers benefits such as time-saving and effective removal of necrotic tissue but also raises concerns like mechanical damage and bacterial propagation into deeper tissue layers [128, 129].

Finally, international guidelines such as those from the WHO and CDC lean toward the use of aqueous iodophor solutions, albeit with a weak recommendation strength [105, 106].

In conclusion, while antiseptic solutions like PVP-I and Betadine have evidence supporting their efficacy in reducing PJI, unresolved issues regarding optimal concentration, comparison with other antiseptics like chlorhexidine, and cytotoxic concerns necessitate ongoing research. Future studies should aim to provide a more nuanced understanding that can guide clinical practice, especially for high-risk patients.

## Perioperative modalities

### Anticoagulation strategies

Total joint arthroplasty (TJA), notably total knee arthroplasty (TKA), is closely tied to concerns surrounding venous thromboembolism (VTE), which encompasses both deep vein thrombosis (DVT) and pulmonary embolism (PE). VTE rates after TKA have been observed to range from 0.9 to 1.6% [130]. This necessitates a well-informed choice of anticoagulation regimen, which effectively combats VTE while minimizing complications, especially prosthetic joint infections (PJI).

A comprehensive comparison has shown the efficacy of aspirin against other non-aspirin anticoagulants for VTE prophylaxis post primary TKA. In a cohort of 6,606 patients, those using aspirin showed a 0.3% rate of developing early PJI within 90 days, compared to a 0.8% rate in the 4,941 patients using non-aspirin anticoagulants. This difference was deemed statistically significant [131]. After adjusting for variables such as age, gender, BMI, and Charlson Comorbidity Index (CCI), aspirin still showcased a statistically significant reduced odds of early PJI [131].

This finding aligns with prior studies such as those by Huang et al., who found that more aggressive anticoagulants like warfarin are associated with higher risks of PJI [132]. Warfarin's administration was associated with up to 13.7 times the risk of PJI when compared to aspirin as VTE prophylaxis [132]. Aggressive postoperative anticoagulation can elevate the risk of bleeding and haematoma formation. This risk further increases the potential of providing a favorable medium for bacterial growth, leading to PJIs. It was previously reported that haematoma formation post hemiarthroplasty for femoral neck fractures could elevate PJI risks [133, 134]. Furthermore, aspirin's effectiveness is not just limited to lowering PJI risks. An et al. in their systematic review found that aspirin offers a low rate of VTE with a reduced risk of major bleeding complications, further bolstering the case for its use as a go-to anticoagulant [135].

A meta-analysis of randomized controlled trials evaluated primary outcomes, including SSIs and PJIs, in various orthopedic procedures (unilateral, primary, and revision TKA, THA, and hip fracture procedures). Secondary outcomes like drug-related adverse events, hemorrhagic wound complications, and persistent wound drainage were also assessed. Enoxaparin and rivaroxaban demonstrated no significant difference in terms of SSI, wound complications, or drug-related adverse events in a significant meta-analysis comprising 12,383 patients. Additionally, there was inconclusive evidence indicating any substantial risk difference of SSI when comparing enoxaparin or fondaparinux to aspirin, even when combined with mechanical prophylaxis [136].

The American College of Chest Physicians (ACCP) and the American Academy of Orthopedic Surgeons (AAOS) have been narrowing down their differences in VTE prophylaxis recommendations over time. These recommendations cover a wide spectrum of anticoagulants, including LMWH, fondaparinux, dabigatran, apixaban, rivaroxaban, warfarin, and aspirin, among others. ACCP's 2012 guidelines do not endorse any specific anticoagulation agent but recommend a balanced approach that considers safety and efficacy, with anticoagulation prophylaxis recommended for at least 10–14 days [137, 138]. These guidelines also leave room for agents like aspirin as acceptable anticoagulants, further adding to the growing body of evidence advocating for its use.

Nevertheless, the choice of anticoagulation should be highly individualized, taking into account patient-specific risk factors. These include age, history of VTE, BMI, and other comorbidities. Several risk stratification protocols have been developed to help tailor anticoagulation regimens to specific patient profiles, although a consensus on a single, validated risk strategy has yet to be achieved [139–141].

In summary, the current evidence strongly suggests that aspirin is not only effective in preventing VTE but also appears to lower the risk of early PJI, making it an increasingly attractive choice for thromboprophylaxis in TKA patients. However, the final anticoagulant strategy should be individualized to each patient's risk profile.

### Blood management

The management of blood during total joint arthroplasty (TJA) plays a crucial role in preventing prosthetic joint infection (PJI) and surgical site infections (SSI). Effective blood management encompasses multiple elements, such as preoperative correction of anemia, thoughtful deliberation on transfusion needs, and the use of both pharmaceutical and mechanical techniques to reduce blood loss [142].

Preoperative anemia, defined as hemoglobin levels below 13 g/dL for men and 12 g/dL for women, is seen in 15% to 33% of TJA patients (144). Patients with anemia face a higher PJI risk, which further elevates by up to 9% with each unit of postoperative allogeneic blood transfusion [143]. PJI is more prevalent in anemic patients at 4.3%, in contrast to 2% in non-anemic ones [25].

The impact of allogeneic blood transfusion on the increased risk of PJI and SSI is well-documented. A 2.88% SSI prevalence was reported among patients receiving allogeneic blood transfusion, compared to 1.74% in those who didn't [144]. Allogeneic blood transfusion increases the risk of PJI by altering immune response and the risk of PJI amplifies with the number of transfused units [145]. To mitigate these risks, strategies like employing hypotensive anesthesia, ensuring meticulous hemostasis, and administering tranexamic acid have been advised to curtail the need for blood transfusion [30]. Notably, Tranexamic Acid (TXA), commonly used to manage bleeding, was also found effective in diminishing infection rates. A plausible reason might be its potential to reduce transfusion rates, which are intrinsically linked to elevated PJI and SSI risks. A comprehensive systematic review and meta-analysis which reviewed 2,259 articles and ultimately included 31, concluded that intravenous (IV) administration of Tranexamic Acid (TXA) has a significant impact on reducing infections in lower limb arthroplasty procedures. The study revealed an overall Odds Ratio (OR) of 0.55 with a 95% Confidence Interval (CI) from 0.49 to 0.62 (P < 0.00001) for decreased infection rates. For Total Hip Arthroplasty (THA), the OR was 0.51 (95% CI 0.35–0.75; P = 0.0005) and for Total Knee Arthroplasty (TKA), it stood at 0.55 (95% CI 0.43–0.71; P < 0.00001). However, topical TXA did not exhibit a corresponding significant effect [146].

Interestingly, the CDC guidelines do not provide a conclusive recommendation on blood transfusion practices for preventing SSI, but they do caution against withholding necessary transfusions [136]. This highlights the complexity of the issue, as blood management practices need to balance the need for transfusion against the risk of infection. Furthermore, contemporary transfusion algorithms recommend more stringent criteria for blood transfusions in ICU patients, both adult and pediatric. Specifically, a transfusion is recommended when the hemoglobin level falls to 7 g/dL or lower. For hemodynamically stable patients who show symptoms like chest pain, orthostatic hypotension, tachycardia, unresponsive to fluid resuscitation, or congestive heart failure, the recommended threshold for transfusion is a hemoglobin level of 8 g/dL or lower. These guidelines are stricter than those in previous studies [147].

In conclusion, an integrated blood management strategy that incorporates the preoperative correction of anemia, judicious use of transfusions, and minimization of operative blood loss is crucial for lowering the risk of SSI and PJI in TJA patients.

## Data Availability

No data sets were generated or analyzed in this review article.
